# Classic and Non-classic (Surrepticius) Scabies: Diagnostic and Treatment Considerations

**DOI:** 10.7759/cureus.7419

**Published:** 2020-03-25

**Authors:** Philip R Cohen

**Affiliations:** 1 Dermatology, San Diego Family Dermatology, National City, USA

**Keywords:** burrow, eggs, feces, ivermectin, mite, permethrin, sarcopetes, scabies, scybala, surrepticius

## Abstract

The morphology of scabies, a mite infestation of worldwide proportion, is characterized by a variety of cutaneous lesions. Patients with classic scabies present with characteristic burrows often located on the web spaces of the fingers and toes. Scabies surrepticius refers to the non-classic atypical presentation of scabies; establishing the diagnosis of scabies in these individuals can be difficult. To facilitate the diagnosis of scabies, criteria have been proposed by the International Alliance for the Control of Scabies (IACS). These criteria are intended for scabies research; however, they can be utilized by clinicians to establish either a confirmed diagnosis, a clinical diagnosis or a suspected diagnosis of scabies. Visualization of mites, eggs or feces is necessary for a confirmed diagnosis of scabies. A clinical diagnosis can be established by observation of either genital lesions in men or burrows or classically distributed classical lesions in individuals with two historic features: pruritus and close contact with an individual who itches and has classically distributed classical scabetic lesions. The clinical features and management of a woman residing in an assisted living environment with a confirmed diagnosis of scabies and a man with a clinical diagnosis of scabies are described. The criteria for the suspected diagnosis of scabies require either one historic feature and typical lesions in a typical distribution or both historic features and the presence of atypical lesions or an atypical distribution of the skin lesions. Once the diagnosis of scabies is established, not only the patient but also close contacts should receive treatment with either a topical medication (such as permethrin 5% cream) or a systemic drug (ivermectin) or both. The number and frequency of treatments are variable; classic scabies typically is managed with a total of two treatments performed weekly to biweekly. Patients with crusted scabies usually require multiple topical and oral antiscabetic treatments in addition to topical keratolytic therapy. Bacterial impetiginization or infection (most commonly by Staphylococcus aureus or Streptococcus pyogenes) can complicate scabies infestation and potentially result in cellulitis, abscess, sepsis, rheumatic fever, rheumatic heart disease and post-streptococcal glomerulonephritis; therefore, in some patients, systemic antimicrobial therapy may be necessary in addition to scabies-directed treatment. In addition to systemic antihistamines, oral and/or topical corticosteroids may be used to provide symptomatic pruritus relief once the diagnosis of scabies has been established and mite-directed treatment has been initiated. The clinician should consider several potential causes (such as inadequate treatment, reinfection, mite resistance, delusions of parasitosis and the development of a new non-scabetic dermatosis) in scabies patients who fail to respond to treatment with a topical or oral scabicide therapy.

## Introduction

Scabies, a mite infestation in humans caused by Sarcoptes scabiei var. hominis, is an ectoparasite dermatosis of global proportion. Indeed, in 2017, the World Health Organization designated scabies as a neglected tropical disease. Identification and subsequent treatment of individuals affected by this condition is essential not only to appropriately manage the patient but also to prevent transmission of the disease to their community [1-4].

A definitive diagnosis of scabies can be established when either mites, eggs or feces (also referred to as scybala) are identified. However, to facilitate scabies research, the International Alliance for the Control of Scabies (IACS) proposed criteria for the diagnosis of scabies in 2018; specifically, they introduced criteria for the confirmed diagnosis of scabies, the clinical diagnosis of scabies and the suspected diagnosis of scabies. The diagnostic criteria have been acknowledged and are likely to be incorporated by heath care providers during their evaluation of patients in whom the possibility of scabies is being considered [5-8].

The clinical features and management of a patient with a confirmed diagnosis of scabies - a woman residing in an assisted living environment who not only had characteristic burrows of classic scabies but also mite-associated lesions of surrepticius scabies mimicking dermatitis - are described; mites were identified on the skin scraping performed from her cutaneous lesions. Also reported is the presentation and treatment of a man in whom the clinical diagnosis of scabies was established based upon the pruritic and characteristic appearing burrows and scrotal nodules from which the microscopic examination of skin scrapings did not demonstrate mites, eggs or feces; he also had mite-related surrepticius scabies lesions mimicking dermatitis. In addition, diagnostic and treatment considerations for classic and non-classic (surrepticius) scabies are discussed.

## Case presentation

Case 1

A 65-year-old woman was referred for evaluation of itching and a diffuse skin rash. She resides in an assisted living residence; one of the residents had been diagnosed with scabies. All of the residents, including this woman, had been treated once with permethrin 5% cream. However, when she developed a generalized pruritic eruption, her primary care physician was contacted and prescribed two doses of ivermectin 12 mg; she had already received the first dose.

**Figure 1:**
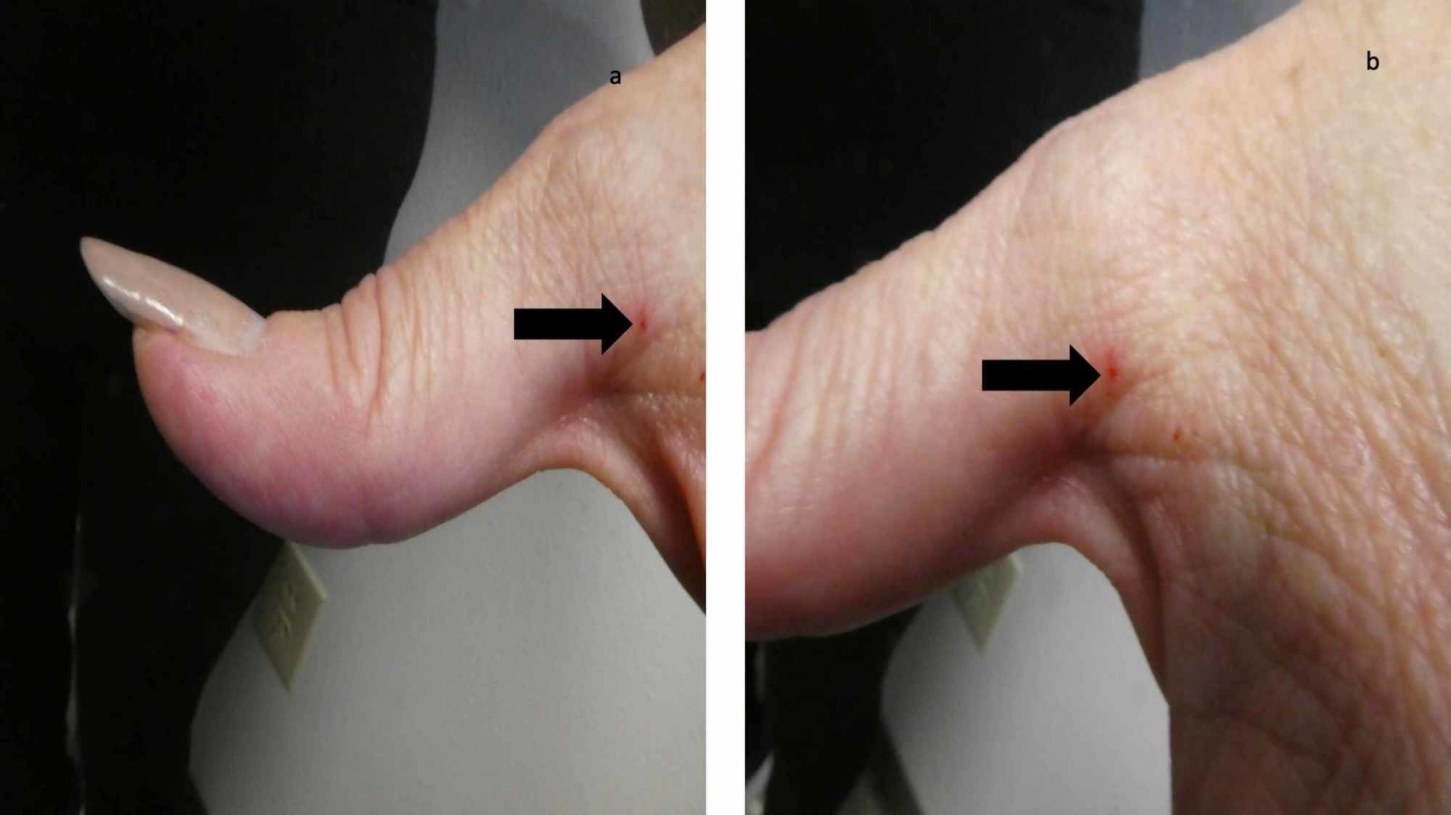
Scabies burrows near the web space between the left thumb and second digit on the hand of a woman with a confirmed diagnosis of scabies Distant (a) and closer (b) views of a burrow (black arrow) on the left hand of a 65-year-old woman. The lesion is red since it was the site of the skin scraping that demonstrated a mite and thereby established a confirmed diagnosis of scabies.

**Figure 2:**
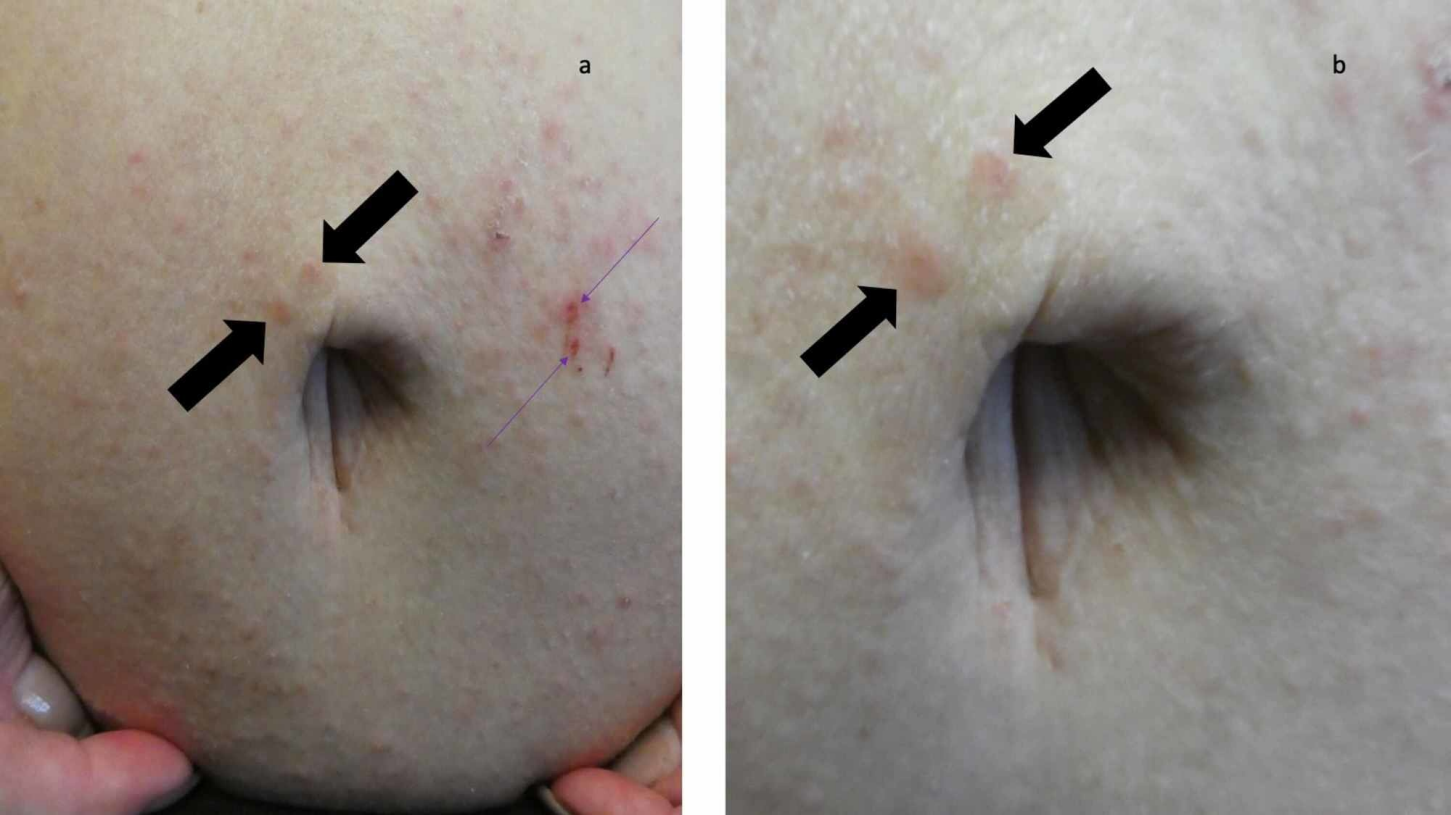
Multiple scabies burrows on the skin surrounding the woman’s umbilicus Distant (a) and closer (b) views of the burrows (black arrows) on the periumbilical skin of a 65-year-old woman. The site of the skin scraping, on the abdomen to the left of her umbilicus (purple arrows), appears red since the epidermis (burrow roof) was removed during the procedure; a confirmed diagnosis of scabies was established since mites were demonstrated on the microscopic examination of the skin scraping and thereby established a confirmed diagnosis of scabies.

**Figure 3:**
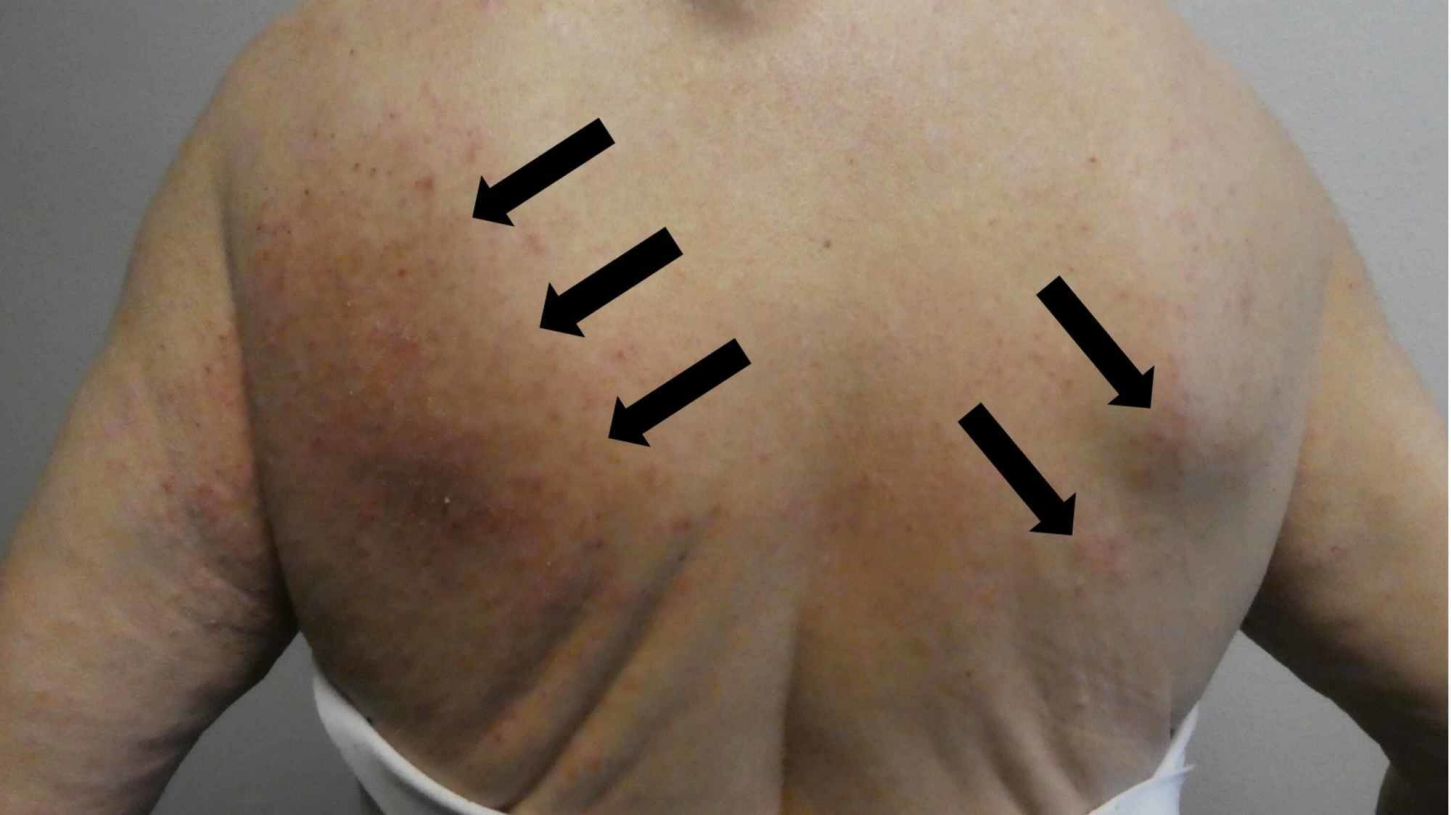
Mite-associated lesions on the woman’s back mimicked dermatitis Individual and confluent, erythematous and excoriated, papules of scabies surrepticius-related lesions (black arrows) mimicking dermatitis.

**Figure 4:**
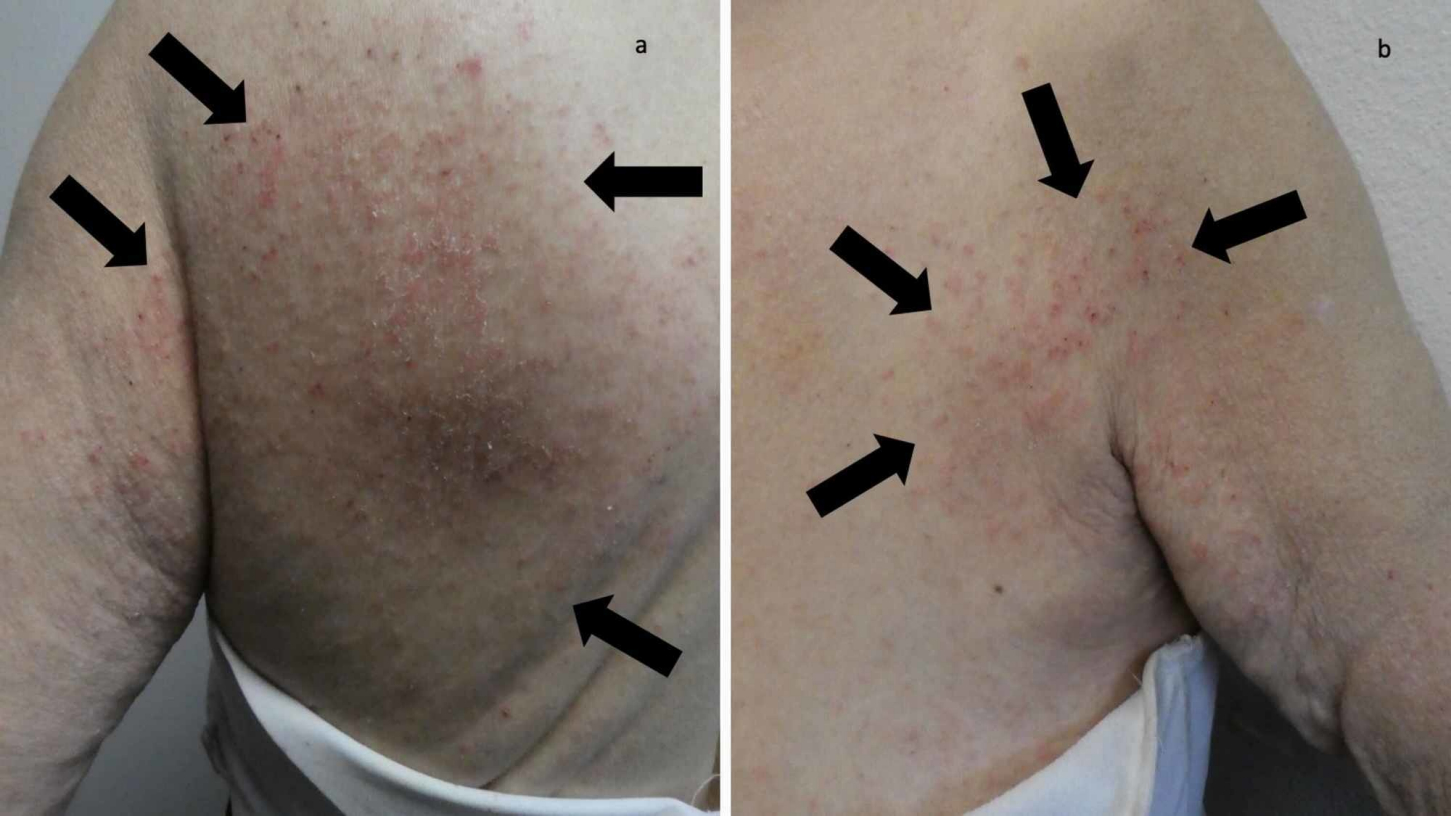
Dermatitis-like scabies lesions on the woman’s back and chest A closer view of the left upper back (a) and the left chest, axillae and proximal arm (b) shows mite-related dermatitis-mimicking lesions of scabies surrepticius (black arrows).

**Figure 5:**
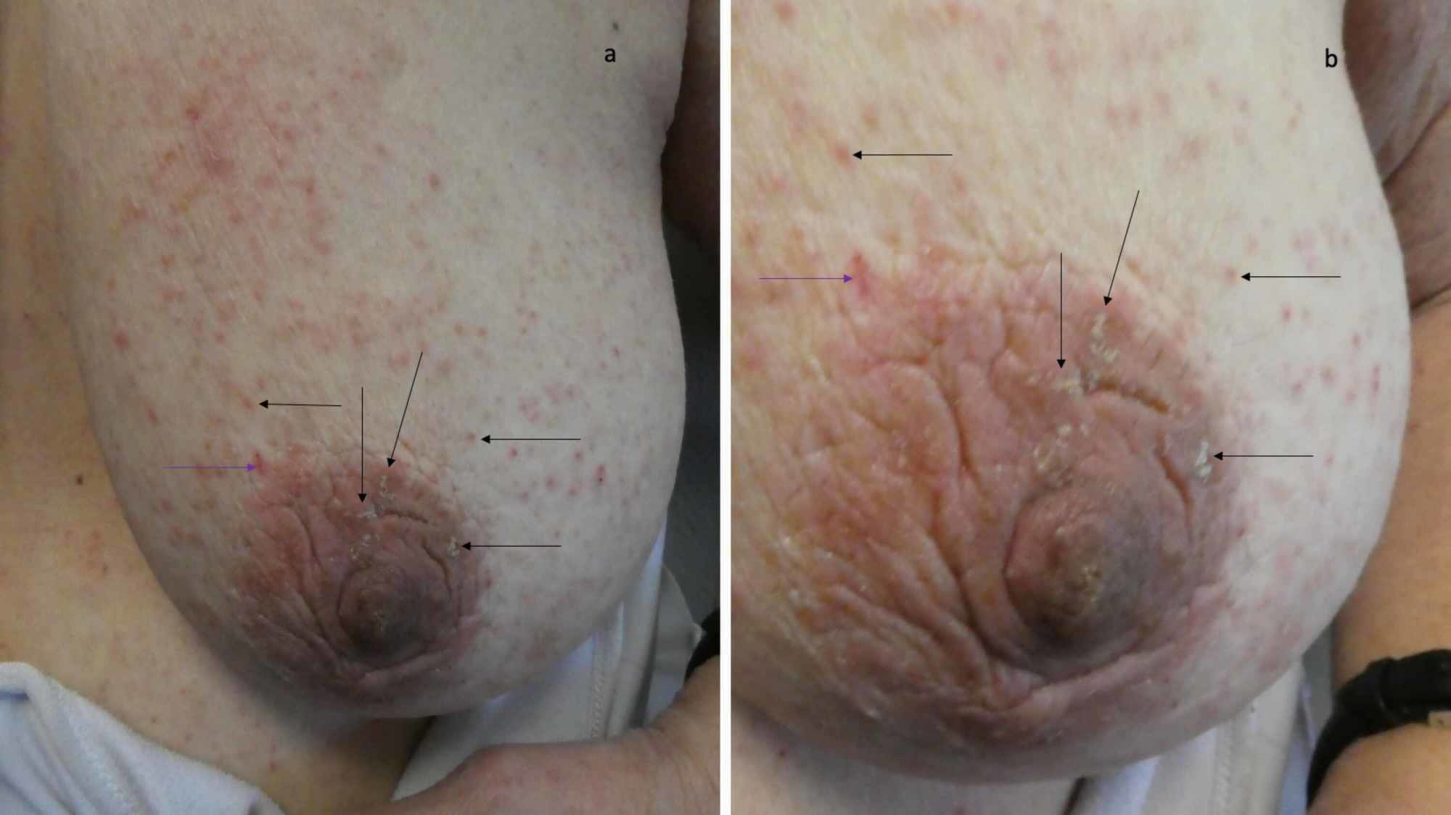
Scabies lesions on the woman’s left areola and breast Crusted burrows (a) and erythematous papules (b) of scabies lesions (black arrows) on the left areola and breast; microscopic examination of the skin scraping site which appears red (purple arrow) showed a mite establishing a confirmed diagnosis of scabies.

A complete cutaneous examination, from her head to her toes, showed linear burrows not only near the web space adjacent to her left thumb (Figure 1) but also around her umbilicus (Figure 2). There were also individual and confluent excoriated papules, mimicking dermatitis, on her upper back (Figure 3); the lesions were greater on the skin overlying her left scapula in addition to also being present on her left chest, axilla and proximal arm (Figure 4). Mite-associated lesions were also present on her left areola and breast (Figure 5).

Microscopic evaluation of a skin scraping, using mineral oil, from multiple sites (including the burrows on her left hand and periumbilical skin, left scapula and left breast) demonstrated mites. Her medical history, the morphology of her cutaneous lesions and the presence of mites on her skin scraping established a confirmed diagnosis of scabies. Additional inquiry revealed that the caregivers at the assisted living residence did not want to upset the patient during her topical treatment with permethrin; therefore, they did not apply the cream to the skin beneath her bra or underpants, the skin around and including her umbilicus and beneath her fingernails and toenails.

Management included topical treatment of permethrin 5% cream (that evening and again in one week) from neck to toes. Importantly, the staff at the assisted living residence was specifically instructed regarding where to appropriately apply the cream. She also received additional doses of ivermectin; since her weight was 145 pounds (67 kg), she was treated with three additional weekly 12 mg doses of ivermectin. In addition, for the symptomatic relief of her pruritus, triamcinolone acetonide 0.1% cream was applied twice daily to any itchy areas.

She was seen for a follow-up examination two weeks later; pruritus was no longer present. Her skin was nearly clear of the previous mite-related cutaneous lesions; plaques were still present on her upper back and areola. A skin scraping from five sites did not reveal any mites, eggs or feces. She would take the fourth dose of ivermectin and continue to apply the triamcinolone acetonide 0.1% cream twice daily to the affected areas for another two weeks.

Another examination two weeks later showed complete resolution of all previous skin lesions. A subsequent follow-up exam one month later did not reveal any new skin lesions. Her scabies infestation was considered to be completely resolved.

Case 2

A 24-year-old man was referred for evaluation of itchy and dry skin of three-month duration. Two weeks earlier, he had noted the development of new nodules on his scrotum. He had no history of close contact with anyone who also itched or had similar appearing lesions.

His primary care physician had initially treated him with doxycycline hyclate 100 mg twice daily for 10 days and triamcinolone acetonide 0.1% cream twice daily to the affected areas. The symptoms and skin lesions persisted. Therefore, his doctor subsequently treated him with cephalexin 500 mg three times daily for seven days and twice daily topical application of fluocinonide 0.05% cream. 

**Figure 6:**
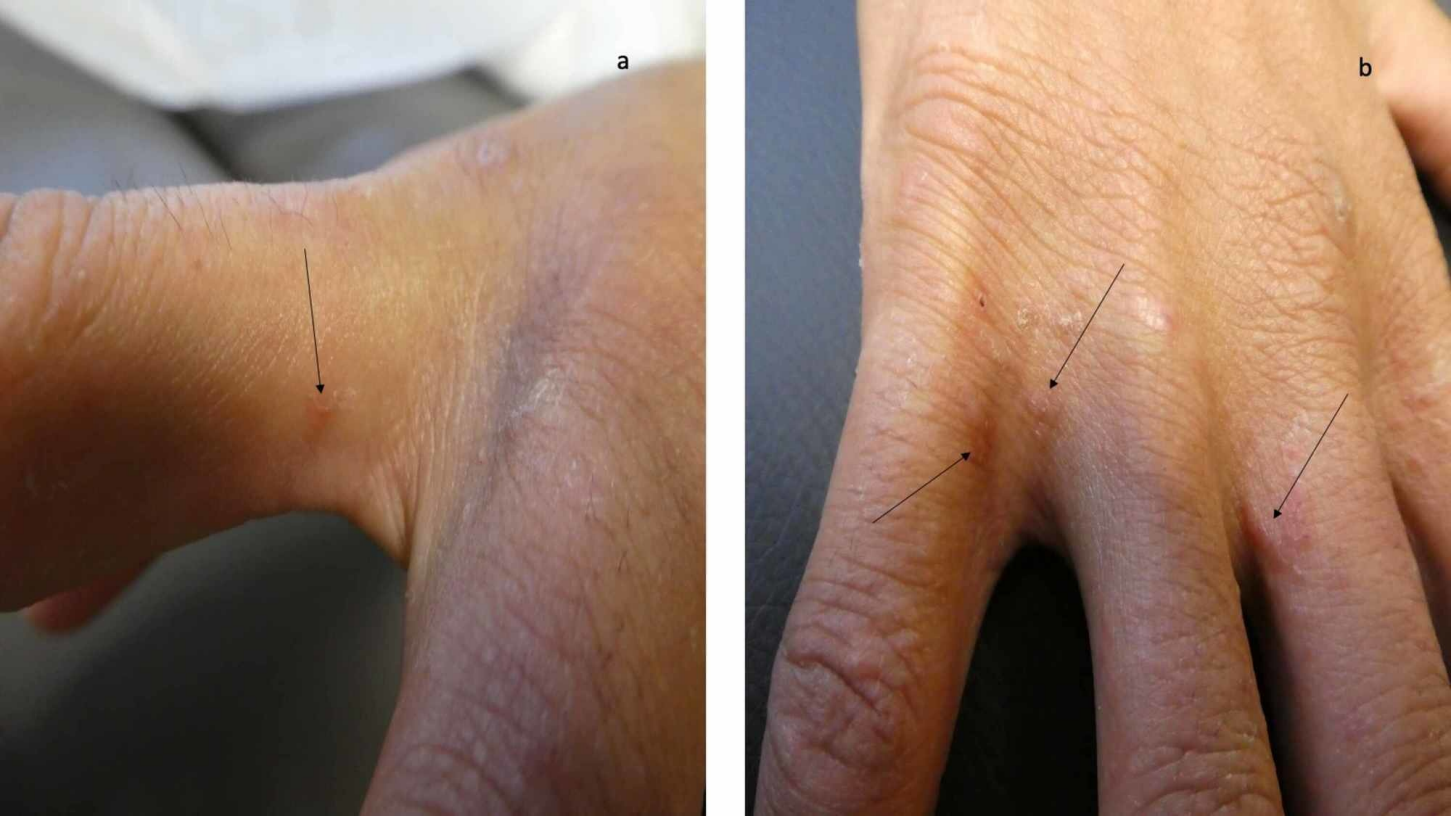
Scabies burrows on the web space between the digits on the right hand of a man with a clinical diagnosis of scabies Burrows (black arrows) are present on the proximal right second (a), third (b) and fifth (b) digits on the hand of a 24-year-old man; they are also present on the web space between his right fourth and fifth hand digits. The skin scraping of the burrows did not demonstrate mites, eggs or feces; therefore, he has a clinical, instead of confirmed, diagnosis of scabies.

**Figure 7:**
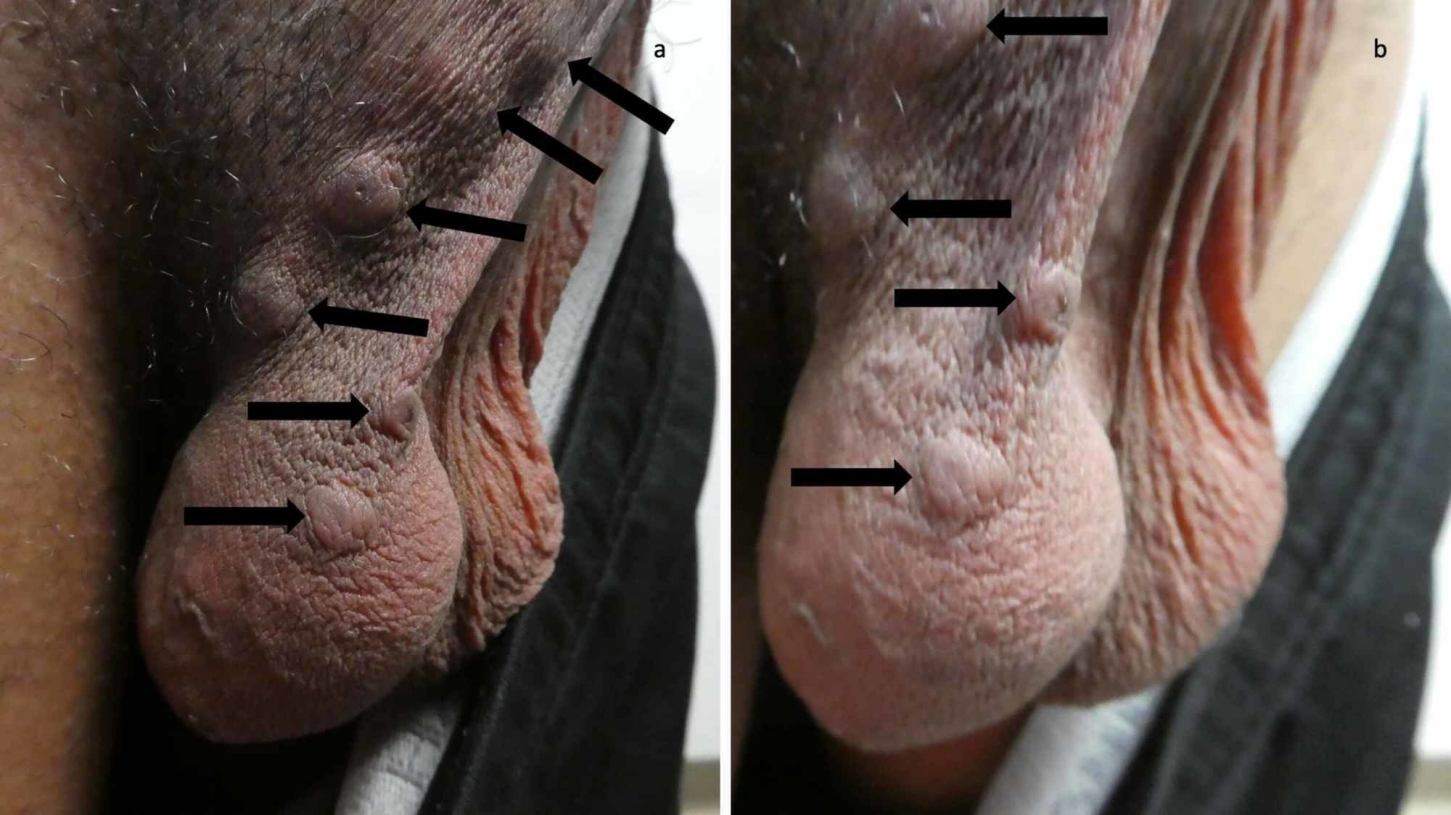
Scrotal nodules of scabies Distant (a) and closer (b) views of the nodules on the scrotum of the 24-year-old man (black arrows). In an individual in whom the diagnosis of scabies is being entertained - yet neither mites, eggs or feces can be demonstrated on the skin scraping from a cutaneous lesion considered to be secondary to the mite infestation - the presence of typical lesions affecting the genitalia such as scrotal nodules establishes a clinical diagnosis (instead of a confirmed diagnosis) of scabies.

A complete cutaneous examination, from head to toe, was performed. Nearly 60% of his body surface area contained eczematous and follicular papules. Close examination of the hands showed burrows on his fingers and the finger web spaces between his digits (Figure 6). In addition, six nodules were present on his scrotum (Figure 7).

An initial and repeat skin scaping, using mineral oil, was performed. A minimum of five sites were evaluated for each of the skin scapings. Microscopic examination of each of the slides containing the skin scrapings did not demonstrate mites, eggs or feces.

Correlation of the medical history, the morphological presentation (scabies burrows and typical lesions affecting the male genitalia) and the absence of mites, eggs or feces provided a clinical-however not confirmed-diagnosis of scabies infestation. His previously corticosteroid-treated lesions (scabies incognito) and dermatitis-mimicking lesions both were also consistent with scabies surrepticius.

Topical and systemic treatment for his scabies infestation was initiated. Permethrin 5% cream was applied from his neck to his toes, including not only beneath his fingernails and toenails but also all cutaneous areas in the genital region (such as his penis, scrotum and the skin between his buttocks) on treatment days 1 and 8. He also received ivermectin 12 mg (based on his weight of 145 pounds or 67 kg) on treatment days 1 and 8.

His pruritus was severe. Therefore, both systemic and topical corticosteroids were prescribed. He took nine days of prednisone (starting at 60 mg each morning for four days and then tapering from 40 mg each morning for three days to 20 mg for the final two mornings) and applied triamcinolone acetonide 0.1% cream to his body twice daily for one week.

His symptoms and skin lesions were significantly improved at follow-up evaluation one week later. The pruritus was gone and the dermatitis-mimicking lesions occupied less than 5% of his body. The scrotal nodules were still present; however, they had begun to flatten.

He repeated the topical and oral scabies treatment the evening of his follow-up visit. He finished the oral prednisone the next two mornings and continued to apply the triamcinolone acetonide 0.1% cream once daily for seven more days. All of his mite-related eczema skin lesions cleared and the scrotal nodules eventually resolved completely.

## Discussion

Classic scabies presents with pruritus that is worse in the evenings. The skin lesions (most commonly burrows) are often located on web spaces of fingers and toes, hands and wrist, and feet and lower legs. In addition, mite-related lesions may be found in the axillae and buttock, the breast and areola of women, and the penis and scrotum of men [8,9].

Non-classic presentations of scabies also occur. In 2017, the term scabies surrepticius was proposed as a designation to encompass all of the atypical presentations of scabies. Subtypes of scabies surrepticius includes not only crusted scabies, but also other variants: bullous, hidden, incognito, nodular, scalp-related and other less common morphologies that mimic dermatitis, dermatitis herpetiformis, ecchymoses, Langerhans cell histiocytosis, pityriasis rosea, prurigo nodularis, systemic lupus erythematosus, urticaria and urticaria pigmentosa [10-13].

The IACS criteria for the confirmed diagnosis of scabies require definitive visualization of mites, eggs or feces. This can be accomplished by identifying a mite on the patient using dermoscopy. Alternatively, viewing either mites, eggs or feces on the individual using a high-powered imaging device (such as optical coherence tomography, reflectance confocal microscopy and videodermoscopy) or on light microscopy of skin samples can establish the diagnosis. A confirmed diagnosis of scabies was able to be established for the reported woman when mites were detected after performing skin scrapings from multiple locations [6,8].

Unfortunately, there are several limitations for health care workers to provide a confirmed diagnosis of scabies. Most facilities do not have the necessary high-powered imaging devices. Also, an evaluation with these devices is not only time consuming but also user-dependent.

In addition, the clinician may not own a dermatoscope. And, similar to the use of imaging devices, a dermatoscope evaluation for scabies is also a user-dependent technique. Indeed, even a dermatologist who has been trained in the routine use of a dermatoscope for inspecting pigmented lesions may have less experience identifying the scabies-associated ‘jetliner with its trail’ sign (which has also been referred to as the ‘delta glider’, ‘delta-wing jet’, ‘jet with contrail’ and ‘spermatozoid’ sign) of the mite head and training burrow [8,14,15].

Finally, a skin scraping of the patient is also dependent on the experience of the clinician to obtain an appropriate sample. Although water, saline or potassium hydroxide solution can be used during the procedure, the procedure is more effectively performed if a more viscous liquid such a mineral oil is used. Sampling more than one location for the skin scraping also increases the possibility of finding mites, eggs or feces. Once the tissue has been obtained and placed on a glass microscope slide, a light microscope is used to examine the sample; however, if the health care facility does not have a light microscope, this procedure cannot be used to provide a confirmed diagnosis of scabies [1,16].

The IACS criteria for the clinical diagnosis of scabies require the clinician to observe either genitalia lesions (such as papules on the penis and nodules on the scrotum in men) or burrows; there does not seem to be an explanation for the paucity of observations of scabies-associated vulvar lesions in women. Alternatively, a clinical diagnosis can be made if the patient not only has skin lesions that are typical in morphology and location but also both historic features of scabies: pruritus and close contact with an individual who itches or has typical lesions in a typical distribution. The IACS emphasizes that a clinical diagnosis of scabies should only be made if other differential diagnoses are considered less likely than scabies. Although skin scapings from multiple lesions did not reveal mites, eggs or feces, the presence of not only burrows on the finger web spaces but also nodules on the scrotum established a clinical diagnosis of scabies in the reported man [6]

Confirmation of a clinical diagnosis of scabies enables the clinician to initiate treatment for the patient and to recommend empiric treatment for family members and other individuals who have experienced either skin-to-skin contact or significant exposure to the patient. Establishing a clinical diagnosis of scabies requires the identification of mite-associated skin lesions; hence, this is dependent on an adequate physical examination of the patient. Some investigators recommend a limited examination (that only includes the hands, feet and lower legs) for research purposes; however, some of these same researchers emphasize that scabies lesions are also identified in area that are normally covered by clothing. Therefore, in an individual in whom the possibility of scabies is being considered, it may be prudent to completely examine the patient from head to toe after all garments, including bra (in women), underwear, shoes and socks, have been removed [2,9].

The IACS criteria for the suspected diagnosis of scabies require either one historic feature and typical lesions in a typical distribution or both historic features and the presence of atypical lesions or an atypical distribution of the skin lesions. Similar to the clinical diagnosis of scabies, making a suspected diagnosis of scabies should only be done if other conditions in the differential diagnosis are less likely than scabies. Antiscabetic therapy may be initiated when a suspected diagnosis of scabies is determined by the health care provider; however, in this setting, it is possible that individuals who appear to have scabies lesions but do not actually have a mite infestation shall receive scabicide treatment [6].

Scabies is usually transmitted by direct skin-to-skin contact and the mite is only able to survive outside the human body for one to three days. Therefore, treatment of the home environment (including fomites such as clothing, bed sheets and covers) is usually not necessary, except in institutional settings, for patients with classic scabies in whom only five to fifteen mites are estimated to be present on their body. However, for the highly infectious patients with crusted scabies - in whom hundreds to thousands of mites are present within the hyperkeratotic scale that is inadvertently being shed - environmental measures to prevent fomite transmission are recommended [4,8,14,16].

Therapeutic interventions available for the management of scabies include topical agents and an oral drug; these can be used either as monotherapy or together. Permethrin 5% cream is the most commonly used topical agent. Although there are no adequate and well-controlled studies in pregnant women, permethrin is considered to be safe for use in pregnancy based on reproduction studies in mice, rats and rabbits (that received oral doses of 200 to 400 mg/kg/day) which revealed no evidence of fetal harm or impaired fertility. In addition, permethrin is approved by the Food and Drug Administration for infants greater than two months of age [8,14,16].

Sulfur (2%-10% ointment or cream) and crotamiton (10% cream or lotion) are also safe in infancy and pregnancy. Benzyl benzoate (a 10%-25% lotion that is safe in pregnancy and is diluted to 6.25% for infants six months to two years of age and 12.5% for children two to twelve years of age) is not available for use in North America. However, benzyl benzoate is used as a second-line treatment in other nations [8,14,16]. 

The number and the frequency of topical scabies treatments remain to be definitively established. Originally, they were only prescribed to be used once. However, therapeutic efficacy is improved when a second course of treatment is performed after seven to fourteen days [8].

Failure to achieve a scabies cure following treatment with a topical scabicide is not uncommon. Indeed, a post-treatment confirmed diagnosis of mite infestation can often be established. Three possible reasons for unsuccessful topical therapy are inadequate treatment, reinfection and mite resistance. Additional reasons for treatment failure also include delusions of parasitosis and the development of a non-scabetic dermatosis [5].

Effective topical treatment requires that the scabicide be adequately applied to the patient. Traditionally, the patient is instructed to apply the medication from their neck to their toes. However, since mites can be located on the scalp (not only in infants, children and the elderly, but also in individuals with congenital or acquired immune deficiency or autoimmune diseases such as dermatomyositis, human immunodeficiency virus, leukemia and lymphoma, systemic lupus erythematosus and trisomy 21), it may be reasonable to consider to also topically treat the neck, ears, face and scalp, especially if there is partial or complete alopecia [16].

In addition, many patients are not physically capable of applying the scabicide to their entire body. Often, they lack the dexterity to reach the mid portion of their back and their feet, including the toes. Also, it is paramount to emphasize that the medication needs to be applied not only to the umbilicus and the crease between the buttock, but also underneath each and every fingernail and toenail; indeed, since mites can be located beneath the nail plate, it might be prudent to have the patient cut all of their nails prior to topical treatment.

The reported woman had a persistent scabies infestation after being treated with topical permethrin 5% cream. However, her caregivers were reluctant to apply the medication to her breasts and to the skin beneath her underpants. Therefore, it was not unexpected that she had numerous mite-related lesions on both of her breasts when she subsequently presented for evaluation. In order to potentially treat the mite infestation more effectively, the patient may return to their clinician’s office or a designated care center, after they have received the medication from the pharmacy, so that trained personnel can appropriately apply the scabicide.

The discovery of scabies after the patient has been appropriately treated (either topically and/or systemically) may be the result of reinfection. Once a patient has been diagnosed with a mite infestation, scabies treatment should be provided not only for that individual but also empirically prescribed for their family and close contacts. A similar approach to management is often initiated for all inhabitants and workers when institutional outbreaks occur and in endemic populations when mass drug administration is utilized as a public health strategy [8].

Resistance of the mite to pesticide therapy, either topical or systemic, is also a potential cause of persistent scabies following treatment. Indeed, an animal model has confirmed the resistance of scabies to permethrin. In addition, resistance of the scabies mite to ivermectin has also been documented in clinical trial patients who failed treatment with this medication [17].

The systemic treatment of scabies relies on the administration of ivermectin, a macrocyclic lactone drug produced from the fermentation products of Streptomyces avermitilis bacteria. Ivermectin is not ovicidal; therefore, a second treatment of the drug (given from seven to fourteen days after the initial dose) is theoretically necessary to kill the newly hatched mites. However, for patients with crusted scabies, either a total of three, five, or seven doses of ivermectin may be given on two consecutive days each week for up to four weeks. Both of the reported patients received more than one treatment with ivermectin [8,14,16].

Ivermectin is not recommended for infants less than 15 kg, children less than five years of age, pregnant women and nursing mothers. The dose of ivermectin for treating scabies is 200 mg/kg (Table 1). Ivermectin is predominantly metabolized in the liver. Its absorption is higher following a high fat meal as compared to a fasting state. Therefore, in order to achieve appropriate absorption, it is recommended that ivermectin be taken with water on an empty stomach [8,16].

**Table 1:** Ivermectin dose for scabies The dose of ivermectin for scabies is 200 mg/kg (which can also be stated as 0.2 mg/kg).

Weight (kg)	Weight (pounds)	Ivermectin dose (mg)	Number of 3 mg pills
15	33	3	1
30	66	6	2
45	99	9	3
60	132	12	4
75	165	15	5
90	198	18	6
105	231	21	7
120	265	24	8
135	298	27	9
150	331	30	10

Crusted scabies is a non-classical (surrepticius), highly contagious, variant of scabies that presents with hyperkeratotic plaques and scales - containing hundreds to thousands of mites - that are diffusely distributed. Many of the predisposing factors noted in individuals with scalp scabies have also been observed in patients with crusted scabies: autoimmune disorders, corticosteroids, immunosuppression, inherited bullous disorders, protein and vitamin deficiency and trisomy 21. The management of a patient with crusted scabies typically requires multiple treatments with both topical and oral scabicides to decrease the high mite burden in addition to the topical application of a keratolytic agent (such as 6% salicylic acid in petrolatum) to promote desquamation of the thick scaly plaques [10,14].

Pruritus in scabies patients may be attributed to a hypersensitivity to mite antigens. Indeed, the patient’s itching may temporarily flare after they receive their initial treatment for the mite infestation. However, if the pruritus persists four to six weeks after completion of scabies therapy, other etiologies for postscabetic itch should be considered: allergic or irritant dermatitis from topical therapy, delusion of parasitosis, inadequate treatment, incorrect initial diagnosis and reinfestation [5,17].

Treatment of mite-associated symptoms, such as pruritus, may be concurrently initiated while the individual is receiving scabies-directed therapy. This may consist of non-sedating and sedating antihistamines in the morning and evening, respectively. Although corticosteroid therapy may be associated with the development of the incognito variant of surrepticius scabies in patients in whom an alternative condition is originally suspected, systemic corticosteroids or topical corticosteroids (as in the reported woman) or both (as in the reported man) may be prescribed once the diagnosis of scabies has been established [8].

The concurrent use of oral antibiotics, such as trimethoprim-sulfamethoxazole, with topical permethrin has been shown to be more effective than either treatment alone for patients with head lice infestation. The lice, which feed on human blood, have a symbiotic bacteria in their gut which is destroyed by the antibiotic; this results in a decline in the lice’s health. Scabies mites mainly ingest tissue fluid and stratum corneum; therefore, in contrast to patients with pediculosis capitis, oral antibiotics have not been demonstrated to be scabicidal [18,19].

However, a potential serious complication of scabies infestation is concurrent bacterial impetiginization or infection (such as impetigo); the most common causative organisms are Staphylococcus aureus and Streptococcus pyogenes (group A streptococcus). Indeed, Streptococcal infection can subsequently result in rheumatic fever (and rheumatic heart disease) or post-streptococcal glomerulonephritis; both bacteria can also result in cellulitis and sepsis. Therefore, coadministration of oral antibiotics with topical and/or oral scabies therapy may be warranted [3,17,20].

## Conclusions

Scabies is a mite infestation of worldwide proportion that can present with characteristic morphological lesions (classic scabies) or atypical non-classic features (scabies surrepticius). The diagnosis of scabies can be elusive; therefore, researchers from the IACS have proposed criteria that can be used by clinicians to establish either a confirmed diagnosis or a clinical diagnosis or a suspected diagnosis of scabies. Treatment of scabies with a topical medication or a systemic drug or both should be initiated not only for the patient but also close contacts once the diagnosis has been established; failure of the patient to respond to therapy should prompt the health care provider to consider inadequate treatment, reinfection, mite resistance, delusions of parasitosis and the development of a new non-scabetic dermatosis in that individual.
