# Correction: Classic and Non-classic (Surrepticius) Scabies: Diagnostic and Treatment Considerations

**DOI:** 10.7759/cureus.c30

**Published:** 2020-05-02

**Authors:** Philip R Cohen

**Affiliations:** 1 Dermatology, San Diego Family Dermatology, National City, USA

In the paragraph directly preceding Table 1 (Discussion section) as well as in the Table 1 legend, the measurement "200 micrograms per kilogram" was incorrectly changed to "200 mg/kg."

In addition, the Table 1 legend text "0.2 milligrams per kilogram" was also incorrectly changed to "0.2 mg/kg."

The text has been updated to include the original language: "200 micrograms per kilogram" and "0.2 milligrams per kilogram" to avoid any confusion. We sincerely apologize for the error.

